# Experiences of wellbeing and resilience among refugee mothers and families in Calgary during the COVID-19 pandemic, and the role of participation in HIPPY, a home visiting program

**DOI:** 10.3934/publichealth.2022036

**Published:** 2022-06-13

**Authors:** Chloe Zivot, Cate Dewey, Meghan Brockington, Chioma Nwebube, Ghaid Asfour, Natasha Vattikonda, Debbie Bell, Sharada Srinivasan, Matthew Little

**Affiliations:** 1 Department of Population Medicine, University of Guelph, Guelph, ON, Canada; 2 The Mothers Matter Centre, Vancouver, BC, Canada; 3 Department of Sociology and Anthropology, University of Guelph, Guelph, ON, Canada; 4 School of Public Health and Social Policy, University of Victoria, Victoria, BC, Canada

**Keywords:** COVID-19, refugee resettlement, Canada, wellbeing, mental health, motherhood, resilience, social support

## Abstract

In order to provide meaningful and effective support to refugees in Canada during the COVID-19 pandemic, as well as during post-pandemic recovery efforts, it is critical to explore the experiences of refugee mothers and families during the pandemic, and to identify sources of resilience that can be leveraged to promote individual and household wellbeing. From November 2020 to June 2021, we conducted in-depth interviews with mothers from refugee backgrounds (n = 28) who resettled in Calgary, Alberta and are currently participating in the Multicultural Home Instruction for Parents of Preschool Youngsters (HIPPY) program. Interviews were conducted virtually using Microsoft Teams; we sought to better understand the pathways and barriers to wellbeing experienced by refugee mothers during the pandemic. The results indicate that the refugee mothers and families in our study experienced widespread disruptions to education and employment and increased motherhood burden, contributing to diminished wellbeing. Mental health was further impacted by heightened levels of worry, stress and social isolation, as well as intense fear pertaining to the spread of SARS-CoV-2. Some mothers reported experiencing barriers to accessing healthcare services and reliable health information during the pandemic. In the face of these challenges, the mothers demonstrated great resilience and identified tangible individual, household and extra-household factors and resources that supported them in coping with the impact of the COVID-19 pandemic. Additionally, our findings suggest that participation in HIPPY played a significant role in fostering the resilience of the participating mothers and families during the pandemic, speaking to the potential of home visiting intervention models in mitigating household hardship during current and future public health crises.

## 1. Introduction

Since 2014, Canada has received over 60,000 refugees through its resettlement assistance programs [1],[2]. Incoming refugees often face social and economic barriers and have increased risk of adverse physical and mental health outcomes compared to immigrant populations in Canada [3]–[5]. Refugees often face multiple challenges during resettlement, including English language and literacy barriers, disrupted or fewer years of formal education, a lack of social support and services (including access to childcare services), increased social isolation, limited access to healthcare, long-term effects of trauma, transportation barriers, unrecognized credentials and difficulty finding employment [6]–[10]. Young families comprise the majority of refugees admitted for resettlement in Canada and navigating these resettlement challenges concurrently during parenthood may result in decreased parental self-efficacy, impacting experiences of wellbeing during resettlement [11]. Further, refugee women, particularly in their role as mothers, may experience resettlement challenges disproportionately, with gender roles, relations and expectations influencing access to health, social, economic and community resources and services [12].

While still limited, emerging research suggests that the vulnerability experienced by many refugee households during resettlement may be compounded by the COVID-19 pandemic. Newcomers, including refugees, face a higher risk of being infected with SARS-CoV-2 [13] due to factors such as over-crowded housing, high-risk or frontline work environments, the prevalence of comorbidities and a limited ability to enact public health safety measures, often due to a lack of access to information or resources [14]–[19]. The COVID-19 pandemic has exacerbated common challenges faced by refugees during resettlement, particularly those of women and young families. Structural and social barriers such as technological barriers to virtual care, cultural incongruity and fear of xenophobia may prevent immigrants from accessing health services during the pandemic [20]–[22]. Distancing and isolation measures have also disrupted livelihoods (i.e., income and food security) and worsened mental health outcomes [22],[23]. In the face of these challenges, newcomer parents, particularly mothers, have reported increased parenting burden, experiences of anxiety and concerns around decreased quality and increased inequity of education [24]; they have also faced a heightened risk of domestic violence and household conflict since the onset of the pandemic [25]. Given the dearth of empirical research addressing refugees' experiences of wellbeing during this unprecedented period, it is critical to develop a deeper and more nuanced understanding of how the COVID-19 pandemic has impacted refugee families in Canada, and particularly women in their role as mothers, in order to inform ongoing policy and future recovery strategies.

In light of resettlement challenges, various social programs seek to assist refugees in Canada with resettlement and integration. Many have rapidly adapted and leveraged pre-existing programs and resources in order to respond to increased demand and public health restrictions during the COVID-19 pandemic. One such program is the Multicultural Home Instruction for Parents of Preschool Youngsters (HIPPY) program. Multicultural HIPPY is administered at the national level by the Mothers Matter Centre (MMC) through partnerships with affiliated local community organizations at 24 sites across Canada. Multicultural HIPPY (hereafter “HIPPY”) is an evidence-based home visiting program focused on parent-involved learning for preschool age children, whereby participating newcomer parents, predominantly mothers, work one-on-one with their Home Visitor (who are often immigrants themselves and previous participants of HIPPY) to support school readiness and increased parental agency. Based on the participant needs, Home Visitors offer additional support and provide guidance regarding the community resources (e.g., food, income and health services) available to refugee mothers and families. Studies have found that HIPPY program participation leads to increased feelings of parental confidence and efficacy, improved literacy and social connectivity [26]–[28]. At the onset of the pandemic, HIPPY rapidly adapted its programming and curriculum to virtual delivery using video-conferencing software. It is possible that involvement in HIPPY may contribute to the mitigation of pandemic-related challenges faced by participating refugee families. Against this background, this study had three objectives. First, to explore the pandemic experiences of refugee mothers in Calgary in order to identify barriers and pathways to individual and family wellbeing during the COVID-19 pandemic. Second, to explore participant and household resilience, which can be defined as “dynamic process encompassing positive adaptation within the context of significant adversity” [29], during the pandemic. And third, to examine the potential impacts of participation in the HIPPY program on fostering resilience during the pandemic, both through its program curriculum and virtual home visiting delivery model.

## 2. Materials and methods

Data were derived from in-depth interviews conducted with mothers from refugee and refugee claimant backgrounds (n = 28) resettled in Calgary, Alberta, Canada, who were participants in the HIPPY program delivered locally by Calgary Immigrant Women's Association (CIWA) during the 2020–2021 program year. The mothers and their families were situated across the city of Calgary, which had a population of approximately 1.3 million in 2020 [30]. In 2016, immigrants (including refugees) comprised approximately 30% of Calgary's population [30]. Our study employed a community-engaged research approach wherein all Home Visitors (n = 11) currently employed at CIWA were invited and ultimately agreed to join the research team as community researchers. In preparation for this role, Home Visitors participated in a seven-hour research training workshop before the commencement of research activities. The training covered topics including research ethics, rigor in scientific research and a detailed overview, discussion, adaptation and finalization of the proposed methods to be used in the project. Our study employed a purposive sampling technique, and upon completion of training, Home Visitors used a script to recruit eligible participants. Eligible mothers included those with formally recognized refugee or refugee claimant status in Canada who were currently enrolled in the HIPPY program offered through CIWA at the time of the commencement of data collection in November 2020.

Interviews were co-conducted by the first author and participants' assigned Home Visitor in their role as community researcher. Interpreters were available for participating mothers who elected to participate in their first or preferred language. Simultaneous interpretation was used during data collection. Researchers asked participants open-ended questions about their experiences of the COVID-19 pandemic, perceived impact on individual and household wellbeing, as well as mothers experiences of participating in HIPPY virtually. The interviews lasted between one and two and a half hours per participant and were conducted using Microsoft Teams video conferencing software. To ensure the comfort of participants facing literacy barriers, the participating mothers provided oral consent to participate in the study, as confirmed in the presence of two members of the research team and recorded in a protected spreadsheet held by the first author. All interviews were audio-recorded, with the exception of one interview, for which detailed notes were taken. Participant quotes have been identified by their assigned numerical identifier in order to protect their identity. This project received ethics approval, including the approval to obtain oral consent, from the institutional review boards at the University of Guelph and the University of Victoria. The methods were co-designed and supported by all members of the research team, leveraging diverse expertise in order to ensure the comfort and safety of participants throughout the data collection process.

Interviews were transcribed by members of the research team from the University of Guelph, as assisted by Otter AI, a secure transcription software. Following transcription, thematic analysis was conducted using NVivo 12. Multiple authors conducted independent analyses of the data as a means of quality control and ensuring rigor. Analyses began with a set of deductive high-level codes determined by the first author. Following independent analyses, inductive codes were presented and major themes were determined by using a consensus-building approach among authors, including those representing community partner organizations.

## 3. Results

**Table 1.** Select household demographic characteristics of refugee mothers (n = 28).

Household Demographic		Households	Range	Mean
*Annual Household Income*				
	0–$20,000	5		
	$20,000–$40,000	12		
	$40,000–$60,000	6		
	>$60,000 No response	1 4		
*Household Size*			3–10	5.14
*Children <18 years per Household*			1–8	3.17
*Number of Children ≤5 Years of Age*			1–3	1.6
	One child ≤5 years	13		
	Two children ≤5 years	13		
	Three children ≤5 years	2		
*Years in Canada (Participant)*				4.75
	≤2 years	3		
	≤5 years	16		
	≤10 years	8		
	>10 years	1		

The mothers' countries of origin include Syria, Eritrea, Ethiopia, Sudan, Venezuela, Iraq, Congo, Nigeria, Angola and India. All but one mother identified as being married at the time of the interviews. The majority of mothers had migrated to Canada as privately sponsored refugees (n = 16), but others arrived as government-assisted refugees (n = 5) and refugee claimants (n = 6), or as blended visa office-referred (n = 1). Select household demographic characteristics, including household income and composition, as well as the participants' number of years in Canada, are presented in Table 1.

The results are organized under two broad topics. First, during the pandemic, the mothers experienced new challenges and barriers to wellbeing resulting from related restrictions, as well as amplification of pre-existing challenges associated with resettlement. Second, the mothers identified critical resilience factors that they were able to leverage in order to support themselves and their families through the COVID-19 pandemic.

### 3.1. Challenges and barriers to wellbeing

Challenges and barriers to wellbeing were categorized into three major themes: (1) disruptions to daily lives and livelihoods, (2) challenges accessing healthcare and (3) mental health challenges during the pandemic.

#### 3.1.1. Disruptions to daily lives and livelihoods

##### 3.1.1.1. Disruptions to child and adult education

Due to the pandemic, education (e.g., school for children, language classes and skill training for parents) shifted online periodically from March 2020 to June 2021. This transition was accompanied by a number of challenges, including families' access to technology (e.g., computers, tablets and Internet) and technological literacy. While some families had access to computers through schools or were able to purchase computers for online classes, several households resorted to using cell phones or other small devices or were forced to share one device across multiple at-home learners.

*“The school was giving away some electronics. But the only thing was if something happens to the electronics or computers, we have to pay $250. So, because we cannot afford it or pay that much money, so we start to use the old computers that we had. So sometimes if we have difficulties or if something [is] wrong with the computer, we have to use small devices like our phones. So, it's a little bit difficult.” (HH14)*

Participating mothers expressed concern that their children were experiencing a decrease in the quality of their education due to technological deficits and the limitations of online learning for immersion and engagement. The mothers felt an increased burden to facilitate their children's education, and some felt that this responsibility was overwhelming or impossible due to low levels of English language or digital literacy. Meanwhile, extracurricular programming, such as optional after-school study hall programs for elementary and secondary school-aged children, were no longer available to students during the pandemic, reducing the support available for children with language barriers and further impeding socialization with peers. Describing the increased barriers faced by newcomer families and long-term implications, one mother shared the following:

*“So many moms are wondering how to help their children at home, because of the language barrier, or because of not knowing about the curriculum, what they are going to learn at school. So those are very challenging for the families that are coming to Canada, because they cannot help their children, they will just say, “okay, go do your homework”. But if the child doesn't know how to do the homework, or if the child doesn't know how to read the book, so it's, it's nonsense to just ask a child, okay, go do your homework, or go read a book* [*...*]. *Helping* [...] *their children is very challenging, they cannot help. So, as the result of that, the children... they will be behind from, when they go [back] to, when they attend school [in-person]. So that really is challenging as well.” (HH29)*

Refugee parents often identified learning English as a priority and crucial to building a life for their family in Calgary. However, many participants faced barriers to their own English language studies during the pandemic (and in the context of a shift to virtual delivery methods) due to added childcare and household responsibilities, technological challenges and inaccessibility of appropriate technological devices. Other barriers to online courses included physical health, such as poor eyesight, migraines and back or neck pain. Participating mothers found it difficult to find time or a quiet environment for their studies with children at home. Some mothers reported having little to no previous experience using a computer and found the learning curve too steep to overcome, particularly when compounded with language barriers or limited experience participating in formal education in their country of origin. As one mother who arrived in Canada less than one year before the onset of the pandemic shared,

*“The COVID-19 issue has made things even much [more] difficult for me. Because even when I want, if I want to get more education, I need to do it online. And I'm not able to do that online because of my level of education. So yes, it's very difficult.” (HH17)*.

##### 3.1.1.2. Impacts on employment, household income and spending

Seven mothers reported temporary or permanent job losses in their households due to the pandemic. Three households did not suffer job losses but experienced reduced household income due to decreased employment hours or reluctance to work due to concern over exposure to SARS-CoV-2. One mother was forced to leave the workforce due to lack of affordable daycare options and an inability to rely on family and friends for childcare support given distancing restrictions. Finally, one mother reported financial insecurity when her husband was infected with SARS-CoV-2 and could not work for two weeks. While many were able to access financial support through Canada Emergency Response Benefit (CERB), employment insurance or other recovery benefits offered through the government of Canada, several families still struggled to cover household expenses.

*“*[...] *when the job is stopped, we just moved to bigger home. So before, we have just apartment [so] the rent is cheaper than here. So, when we arrived everything is different, expensive. So [then] he has to stop the job. Okay, the government give us but it's not enough because the government gives us $2000, but the rent and utilities is $2000, so [for] everything else I had to take out [a] loan, for insurance, food, diapers, everything. So yeah, everything is too bad.” (HH04)*

Household financial insecurity was further exacerbated by increased expenses during the COVID-19 pandemic. For example, early in the pandemic, several households were forced to purchase laptops to support online learning. One mother noted the increased cost of food and household items at the onset of the pandemic.

##### 3.1.1.3. Experiences of motherhood

Participating mothers had between one and eight children in their household, with an average of three children per family. Some mothers reported increased support from their partners during the pandemic due to reduced responsibilities outside of the home. More frequently, however, mothers expressed experiencing increased parental burden throughout the COVID-19 pandemic due to the limited support from non-household friends and family and the suspension of community support and programming systems. Mothers from larger families found it particularly difficult to keep young children quiet and occupied while other family members participated in online education. Some mothers were forced to be the sole caregiver when their partners had to self-isolate due to workplace exposure to SARS-CoV-2. A single mother in our study described the stress and difficulty of shopping for groceries and other necessities while having nobody to provide childcare yet not wanting to increase her children's exposure to the virus by bringing them along. Mothers frequently reported prioritizing their children's mental and physical wellbeing over their own, leaving little time for their own care:

*“Because I'm afraid about her (daughter) mental [health], so I have to take whatever I [...], like if I have a plan to cook my lunch, I just forget about my lunch and then I have to take care of things [for her] and then just [pick up] drive-through [meal] for me and then [...], yeah, I just sacrifice my things for her mentality.” (HH03)*

Another mother described the increased parental burden due to limited opportunities for socialization as a result of public health recommendations regarding social distancing:

*“So, the impact of COVID-19 is very huge, especially for children, as well as for moms. Because for example, me, I always enjoyed when I go to [main office of community service provider], because meeting new friends or the children will be playing, that's so enjoyable for me. But staying at home with the children is very stressing for the children as well because they cannot meet a new friend, they cannot play with other children. So it took a lot of energy for moms to be staying at home, so it's very tiring.” (HH18)*

#### 3.1.2. Challenges accessing healthcare

During the COVID-19 pandemic, access to health services was limited by a number of factors, including fear of exposure to SARS-CoV-2 in health facilities, limited access to reliable health information, widespread misinformation, quarantine procedures after exposure to SARS-CoV-2 by a household member, a loss of medical benefits due to unemployment and health system delays due to pre-emptive cancellation of non-mandatory surgeries and referrals. Those who sought healthcare during the pandemic often felt unsatisfied with the care they received. One mother felt she did not receive support in resolving her debilitating migraines after she was told to undergo further testing but then received no follow-up care. Some mothers mentioned feelings of discomfort during medical appointments due to abrasive interactions with providers and the added difficulty of communicating while wearing masks, which amplified language barriers and prevented lip-reading. One mother reported no follow-up contact or care after receiving what was ultimately a positive COVID-19 test. This participant reported self-isolating until she was able to call and confirm that her test was positive, at which time she was provided the relevant information to self-isolate safely and effectively.

#### 3.1.3. Mental health challenges during the pandemic

##### 3.1.3.1. Social isolation

Mothers described feelings of sadness and discomfort as a result of increased social isolation due to distancing requirements. Attending in-person classes, visiting and celebrating with family and friends and attending religious gatherings were identified as factors that contributed to wellbeing during resettlement. Restrictions on such activities posed a challenge to mental health. Several mothers were deeply disappointed when travel restrictions forced them to postpone travel or sponsorship plans in order to reunite with extended family. For some, the pandemic exacerbated experiences of isolation already common during resettlement:

*“Already we don't have like really this social life in Canada and with the pandemic it's getting worse. We already we don't have it as in back home, we don't see usually many people, and now we don't see any.” (HH10)*

##### 3.1.3.2. Stress and worry

The increased social isolation experienced during the COVID-19 pandemic contributed to heightened stress and worry, with one mother sharing,

*“Maybe because I am not able to go out and do whatever I want on my own. So I feel, sometimes I feel stressed and get nervous so quickly.” (HH17)*

The mothers described several sources of stress and worry that were related to the COVID-19 pandemic, including an inability to socialize (which normally serves as a stress-coping mechanism), delays in adult education and employment, reduced household income, increased household expenses and distress experienced by their partner or children. In reference to her husband's anxiety throughout their prolonged refugee claimant process and the supportive role of her brother-in-law, one woman shared the following:

*“[Her husband's brother] always encourages us and sometimes we go out, even before COVID, when [her husband] got [anxious] [...] he was [worrying about things], and [...] sometimes he always tell him, okay, brother, let's go somewhere. Let's just have little breeze, let's go out to talk* [...] *and all that. And it's really helped him. Yeah, it's helped him a lot. Me, myself, when my husband is down, I'm always down. But I always want to encourage him. I won't show him that I'm down, but I'm down.” (HH12)*

Several mothers discussed the implications of heightened stress on their family's economic and social resettlement outcomes, with one mother sharing the following:

*“Like for example, it's like a circle, so the English has been stopped, so the good job needs English and they cannot do English because he's tired and he's tired because, you know (increased stress), it's like a circle, it never ends.” (HH23)*

##### 3.1.3.3. Concern for children's mental health

The mothers also expressed concern about the wellbeing of other family members, particularly their children. The mothers felt that their children became irritable, sad and confused about their inability to attend school, visit with friends and participate in extracurricular activities. Some children found the lack of structure in their daily lives to be disorienting:

*“Before [in Spring 2020], the online school was very flexible. And they start to sleep almost all day. And they[...] like, the lack of routine affects the kids, you know? At the beginning, I say okay, wake up and go to bed, “for what?” they say, “for what?”, it's like they don't have any reason to wake up. You know? And the teacher, maybe it was because maybe they don't know how was the mental health of everyone.” (HH05)*

##### 3.1.3.4. Fear of COVID-19

Intense fear of the SARS-CoV-2 virus, particularly in the early months of the pandemic, contributed to social isolation and high levels of stress. Many mothers believed it was not safe to go outdoors for walks in their communities or visit with others, even in socially distanced settings. Participants also worried about the health of aging parents and other family members, particularly in instances wherein extended family resided in countries with fragile or limited healthcare systems. As shared by one participant,

*“All the time, I feel sad. All the time, if I do something special, I'm not[...] I'm not sure what I feel[...]. Always I feel something not good. Not because[...] what's happened about[...] about the job because no money—no, no, not like that. But yeah, everything is* [...] *not good. Every time[...] every day I heard someone is death from the corona. Every day I heard someone is pain, has a pain. So everything around us is not good. So that's why I can't feel good.” (HH04)*

Balancing the fear of exposure to SARS-CoV-2 and the requirement to meet household needs was a challenge for some. One mother expressed the frustration and helplessness she felt due to her husband's refusal to return to work due to safety concerns, despite what she perceived to be a low-risk occupation, in the face of financial insecurity:

*“Yes. After long time, he [re]start job, oh my goodness. But why [wouldn't he return to work], because still everyone start job. Many people, they working like at the restaurant, something that never[...] they meeting with people that never stop, [they haven't stopped work to] stay home. Why he cannot work? Because he's working alone, he doesn't meet many people. Also, you must go [to your] job. [Supporting the family] is difficult without job, without money.” (HH25)*

Finally, fear of the SARS-CoV-2 virus may have been amplified for recently resettled families working through the trauma and mental health implications related to having recently escaped a violent conflict or war in their countries of origin. One mother told us the following about another recently arrived refugee family in her community:

*“And then the other family who really suffered from the war in their country, and then they just were seeking for a safe place to live with their kids. And when they arrived to Canada, the pandemic started and then, like, they said,* [...] *we did not die from the war* [*...*] *war in our country, we came to Canada to die here of the* [...] *pandemic* [*...*] *So they were really scared... this family, [it] really affected their mental health, the pandemic and staying at home when [they are] still new to Canada.” (HH10)*

### 3.2. Resilience in the face of the COVID-19 pandemic

The mothers exhibited strength in the face of compounded adversity and emphasized resilience factors that facilitated coping with challenges during the pandemic. The resilience factors included those associated with physical or lived environments, social interaction and support and mental attitudes and perspectives. In addition, participation in HIPPY emerged as a critical support during the pandemic.

#### 3.2.1. General resilience factors

The participants identified several resilience factors associated with their environments. For example, for some households, access to a more spacious living environment (e.g., living in a house instead of an apartment) made it much easier for family members to participate in online classes without interruption. This also allowed for the safe and effective self-isolation of family members exposed to the SARS-CoV-2 virus. Yet, this is not to say that access to adequately spacious housing was experienced by all participants. Spending time outdoors, particularly taking walks around the neighborhood, helped to mitigate stress, worry and a sense of isolation for many mothers:

*“Yes. But it was very, very, very, very difficult situation because I, I like to go outside* [*...*] *with the fresh air, me and the kids. What they tell me first thing, first time corona, everyone is staying at home, closing everything. My mind it was crazy. Yeah. But after that, when I start* [*...*] *when I saw people wearing a mask going for any part, a walk, something like that, that... I stand again.” (HH25)*

Social and economic support systems were important sources of resilience for many households. Connecting with friends and family over video calls mitigated feelings of isolation and stress. Having older children who were able to help mothers navigate learning technology, as well as husbands who were active in supporting children with online schoolwork, helped to relieve the parenting burden and barriers to education for some mothers. Economic support systems (e.g., CERB) and social services (e.g., emergency food services) were critical in supporting household wellbeing for many households who faced livelihood disruptions.

Finally, many mothers seemed to draw resilience from holding optimistic mental attitudes and perspectives. Several mothers discussed the power of keeping a positive attitude and chose to find silver linings in the face of challenging situations, such as the increased amount of time spent together as a family unit and, in some cases, the rare opportunity for a break from demanding work schedules. In response to her husband's job loss, one mother shared the following:

*“Yeah, because when he received the EI [employment insurance] in the beginning, he was worried, [and] me too, we say, I say, oh my goodness, here is hard to find a job...but [on] the other hand I say [to husband], “that's good (job loss) because you don't take vacations. You have been four years working [without taking any time off].” (HH02)*

Many coped with challenges during the pandemic by maintaining a perspective on the country's collective experience in struggling with the pandemic, instead of focusing on their own personal or household's situation:

*“Sometime, [life] it's good, sometime like I am going work, I'm gonna be okay, [but] some time you're not working, it's gonna be hard... [but] then [I remember or think] it's not myself [only], everybody in the world have problem from COVID-19, and then it's okay.” (HH01)*

While some mothers experienced challenges associated with learning new technology in order to access education, medical resources, assistance and other services, many also embraced the need to develop technological literacy.

*“I feel comfortable with the technology right now because we need to adapt to these* [*...*] *this new normal, no?” (HH05)*

#### 3.2.2. HIPPY as a source of resilience

In March of 2020, HIPPY activities in Calgary and across Canada transitioned to virtual delivery after two weeks of disruption. The mothers and Home Visitors then continued to meet weekly using Microsoft Teams. According to the interviewed mothers, participation in HIPPY contributed to the mitigation of challenges resulting from the COVID-19 pandemic. In the process of transitioning the program to virtual delivery, HIPPY Home Visitors helped mothers learn to navigate various computer applications and virtual communication platforms and gain online access to services and support. HIPPY also provided participating mothers with access to low-cost tablet, which not only assisted mothers with participating in HIPPY, but played an important role in mitigating technological barriers to education and social support, as children and parents alike used the tablet to access online schooling and communicate with family and friends:

*“At the beginning, I didn't have any laptops, I was only studying on my cell phone and it's been really hard. So that tablet has been an improvement because it's bigger. So when it's bigger, it gives me more access, because I used to like put the cell phone in front of my eyes all the time and used to make my eyes [strained]. So bigger is better. So yeah, it's been good.” (HH26)*

Seven mothers discussed the benefits of having the materials and activities provided by HIPPY to engage and occupy young children, which minimized disruptions to at-home learners and helped mitigate exhaustion or burnout from added childcare responsibilities. Free children's books were distributed through HIPPY, and they were helpful to mothers when public libraries were closed. In the absence of pre-school and affordable daycare, the mothers expressed gratitude for the teaching and literacy skills they developed through HIPPY. These skills provided the mothers with confidence in their abilities to support their children's learning, which in turn allowed mothers to better cope with the emerging expectation that they play a more active role in facilitating at-home learning for their children during the pandemic.

*“So it's very, extremely helpful having HIPPY program. Because at this pandemic, how do I bring books for my children. While I'm at home, HIPPY will provide me with some books and activities. So I will do the fundamental things for my children, the basic things that they can have to learn. So I'm helping my children at home without going outside. Because the libraries are closed, I cannot bring books for my children. But with HIPPY being providing this books and some extra activities was very helpful.” (HH29)*

Home Visitors played a crucial role in providing information to refugee families during the pandemic, including information about available government and community services and support systems, news on pandemic restrictions and information on the nature and spread of SARS-CoV-2 in the face of widespread fear and misinformation. They also supported the mothers in accessing these support systems by assisting with paperwork and online form submission under circumstances wherein the technological and language barriers were high:

*“I can find* [*...*] *now I know like I have a support. If I have any challenge, any problem I know like who to go and talk to. This is [Home Visitor].” (HH10)*

Finally, Home Visitors, as well as online group meetings organized to connect HIPPY mothers, provided a crucial social connection throughout the pandemic, particularly for those mothers who had resettled in Canada in the year or months leading up to the onset of the pandemic:

*“I think this, the HIPPY program has been so much helpful for me. Actually, I have never talked to anyone in this country, I just talk to my friends back in Sudan or in Egypt. I've never talked to anyone here. So through HIPPY, I'm able to talk to other people. And so it just make me, give me a relief when I say something what, about what I feel inside. It's a big relief for me. So it's really helpful. Even if it's online, I like it.” (HH17)*

## 4. Discussion

Refugee mothers and families experienced new and amplified challenges during the COVID-19 pandemic, which influenced their mental and physical wellbeing during resettlement in Calgary. Specifically, increased barriers to education and employment resulted in stress and worry in parents. The mothers reported increased parenting responsibilities concurrently with heightened isolation, which contributed to perceived declines in mental health. Yet, the mothers demonstrated resilience in responding to these very challenging circumstances and discussed resilience factors at the individual, household, community and structural levels that supported them in coping with the challenges associated with the pandemic. Participating mothers shared insights into the role of HIPPY in mitigating new and amplified resettlement challenges during the pandemic. These findings provide insight into the potential for home visiting models of social programming to dynamically respond to and meet the needs of refugee families in the face of public health crises or other external shocks.

Participants expressed concern regarding the quality of their children's education following the transition to virtual delivery methods. Insufficient access to technology, particularly in households with multiple at-home learners, was common. Participants discussed challenges associated with the expectation that mothers assist their children in online learning activities, which for many was impeded or made impossible by low levels of technological literacy and English language proficiency. Such findings align with those in emergent literature that examine refugee and immigrant parents' and students' pandemic experiences [24],[31], as well as those of refugee parents in other high-income resettlement contexts [32], and mothers within the broader Canadian population [33]. As discussed by Guruge and colleagues (2021), normative assumptions of household privilege (in terms of access to technology and the capacity of parents to assist with education) informed the rapid shift to virtual education, yet do not reflect the reality of many newcomer families in Canada [24] or beyond. As a result, refugee students may find themselves marginalized in their virtual classrooms, exacerbating inequities in education and contributing to disparities in learning outcomes. Participating mothers were also concerned about the impact of virtual delivery on their children's socialization and acculturation. These concerns are well-founded, as existing literature has established the school environment as a facilitator of social integration during resettlement, which is critical to children's wellbeing [34]–[36].

In accordance with pre-pandemic resettlement literature, refugee mothers recognized English language proficiency as a crucial determinant of wellbeing during resettlement [12],[37],[38]. Technology and language barriers, as well as increased parental burden in the face of school and daycare closures, prevented many mothers from engaging in or continuing their studies at the onset of virtual delivery. This finding is consistent with those in recent literature [24],[39], and these barriers may impact women disproportionately [39] as a result of the uneven burden of additional childcare responsibilities during the pandemic. Johnston and colleagues (2020) observed Canadian women's weekly hours of childcare obligation to rise from a pre-pandemic average of 68 hours per week to 95 hours during the pandemic, with women allocating 2.5 times more hours per week to childcare responsibilities than men [40]. Disruptions to adult learning, particularly English classes, may have a significant impact on the mental and physical wellbeing of refugee families. In the pre-pandemic context, language acquisition and skills are widely cited as a determinant of refugee employment in the Canadian and other high-income resettlement contexts [41], which in turn acts as a determinant of mental health [37],[42]. Further, previous studies suggest that attending language classes plays a role in mitigating isolation during the resettlement process [23],[43]. Language barriers may also impede access to healthcare and other crucial services during early resettlement [44]–[46], particularly for women [12]. These challenges may also limit access to reliable health information, potentially contributing to the fear and mistrust of mainstream sources of information, spread of misinformation and increased risk of SARS-CoV-2 infection [47].

Our findings suggest that the COVID-19 pandemic and related social and economic disruptions influenced the mental health of participating refugee families in ways that align with emergent literature [22]–[25]. Participating mothers were concerned about their children's mental health and social development in the face of increased isolation due to school closures and distancing requirements. Mothers also reported increased isolation among themselves, partially due to increased childcare responsibilities. Distancing requirements and a fear of the SARS-CoV-2 virus limited women's mechanisms for coping with isolation and their mothering burden, such as taking their children to the park, visiting with friends and family and receiving childcare support from their extended family and social network. Increased unemployment and household income insecurity were major sources of stress for families. Mothers worried about finances, and several expressed concern for their husbands' mental health, which they perceived to suffer due to job loss and household financial strain. These findings highlight the role of pandemic disruptions in amplifying pre-pandemic risk factors for refugee mental wellbeing, including isolation, unemployment and having a low income and the absence of or separation from family [48]–[49].

Further, our findings support previous literature on financial insecurity in refugee families, showing that experiences differ between mothers and fathers as a result of pervasive parental and gender roles [12],[50]. Participating mothers prioritized their children's needs before their own, something that is well documented in pre-pandemic literature [3],[51],[52]. Participants spent a considerable amount of time keeping their children occupied with in-home activities during the pandemic [24],[33]. As a result of increased household and parenting responsibilities, mothers felt they had little time for their own self-care [24],[33]. Such an impact may have contributed to an observed increase in maternal depressive and anxiety symptoms in Canadian mothers during the pandemic [53],[54]. The literature suggests that the gendered impact of the pandemic includes a disproportionate increase in parenting burden on the mothers. Johnston and colleagues (2020) found that Canadian women reported worse overall mental health compared to men in households with children aged under 25 years during the pandemic [40]. An increased burden on mothers may also influence mental healthcare-seeking behavior; for example, Cameron and colleagues (2020) reported that, among mothers with clinically relevant depression or anxiety during the pandemic, 34.5% identified limited time and energy as barriers to accessing mental health services [54]. Evidence of disproportionate experiences of parental burden and decreased mental health among women [40], with potential impact on decreased access to mental health services [54], was reported in reference to the wider Canadian population versus newcomer-specific populations, suggesting that pervasive gender norms translate to increased motherhood burden, irrespective of cultural context. Yet, our findings suggest that the increased burden may be exacerbated for refugee mothers, as those in our study simultaneously faced the prevalent resettlement challenges highlighted in our results, including language, technological and structural barriers to information and social support.

Participating mothers showed strength and resilience in the face of new and compounded resettlement challenges during the COVID-19 pandemic. Resilience can be understood as a “dynamic process encompassing positive adaptation within the context of significant adversity” [29]. The mothers in this study reported several factors that led to increased resilience, including receiving emotional, childcare and technological support from partners and children, spending time outdoors in socially distanced settings and trying to keep a positive or optimistic perspective. These findings align with the existing literature pertaining to experiences of resilience in the context of the COVID-19 pandemic [24],[55], as well as with pre-pandemic literature on resilience in refugee mothers during resettlement [52],[56],[57]. Additional coping mechanisms included adopting a global perspective and collectivist attitude (i.e., framing one's own challenges against the backdrop of the pandemic being a global event affecting everyone), which was a common practice to situate, contextualize and lessen one's perceived struggles. While there is little research substantiating this finding in resettled refugee populations or the wider Canadian population, a recent study by Vignoles and colleagues (2021) found that shared social identification with humanity predicted greater mental wellbeing and lower depressive and anxiety symptoms among an adult population in the United Kingdom [58].

Seminal works in resilience literature call for a shift in the conceptualization and analysis of resilience from the perspective of individual protective factors toward a conception of resilience as a collective process that includes community and structural forces [29],[59],[60]. Termed “collective resilience” [56], this broadened conceptualization establishes resilience as a continuous process that incorporates the individual, family and wider social environments, and recognizes that resilience is not inherent to individuals or households but is built up through support systems [59], such as those provided by government and non-governmental organizations. Indeed, our findings underscore that extra-household support systems are critical to refugee mothers' and families' wellbeing during the COVID-19 pandemic. Financial support from the government, through programs such as CERB, was cited by our participants as key to mitigating stress amid widespread layoffs, echoing broader findings on the role of CERB in supporting Canadian families. Prime and colleagues (2020) have suggested that the level of risk posed by financial hardship to caregiver wellbeing will likely depend on the families' pre-pandemic economic situation [55]. Given the prevalence of low-income status among refugee households both in our study (See Table 1, which shows that the low-income cut-off for a family of five in a city the size of Calgary in Canada as of 2019 was $47,148.) [61] and in the wider refugee population [62], it is possible that financial support systems such as CERB are particularly crucial in mitigating risks to mental health in refugee populations in response to economic shocks.

Social support is a critical determinant of health during refugee resettlement in the Canadian context and beyond [5],[63]. Formal social support programs with refugees in the pre-pandemic context have encouraged social integration, contributed to improve physical and mental health and facilitated increased household resilience in the face of resettlement challenges [64]–[66]. Our study explored the impact of participation in HIPPY, a parent-centered home visitation model of social intervention, on wellbeing and household resilience during the COVID-19 pandemic. Our findings suggest that participation in HIPPY was an important contributor to household resilience among refugee families. Through the HIPPY curriculum, families were provided activities, crafts and books to keep their children occupied [24],[33]. Tablets provided to mothers were useful for online learning outside of the HIPPY curriculum, such as virtual schooling for children and language classes for the mothers. Tablets were also used to maintain social contact with family and friends. The fact that the tablets were widely reported by mothers as instrumental to maintaining consistent access to social programming and networks indicates wider implications for current and post-pandemic service provision. Should virtual delivery become more common, it will be critical to proactively address the barriers to participation some may face. Further, outside of their use for participation in programming, the provision of tablets to refugee families for general use during resettlement may have the potential to mitigate social isolation and promote improved access to information. The increased English literacy and teaching skills fostered through HIPPY programming provided the mothers with greater confidence to support their children's education, helping to mitigate the stress and guilt associated with concerns for their children's quality of education. Improving parental self-efficacy is a protective factor for mothers' mental health [67] and, in turn, children's and family wellbeing [47],[55], to the extent that the caregiver and children's wellbeing are “inextricably linked” [47].

A unique aspect of the HIPPY program is its delivery model, in which mothers meet weekly with their Home Visitor (typically in mothers' own homes) for the duration of each program year. The program is offered to participants for multiple years, and mothers often work with the same Home Visitor for the entirety of their time in the program. The mothers and their Home Visitor often develop rapport and comfort with one another. As a result, Home Visitors develop a nuanced understanding of their clients' needs and often become a trusted source of information and point of first contact when challenges arise in the household. Our findings suggest that this unique model of service delivery was well-suited to responding rapidly to the specific needs of each mother during the pandemic. The HIPPY program transitioned to virtual delivery with little interruption to the scheduled programming. This was feasible in large part due to the pre-existing client-provider relationships and one-on-one delivery format. In other words, Home Visitors were already very familiar with the mothers' specific individual and household contexts and language and communication barriers, and could proactively arrange transition support according to the mothers' needs. As a result, HIPPY clients experienced a high degree of continuity of support during the dynamic, confusing and acutely stressful early months of the COVID-19 pandemic. As programming continued throughout the pandemic, Home Visitors were able to tailor their support to each mother and household, providing referrals for food and mental health services, assisting with applications for emergency government support and supporting the mothers in improving technological literacy skills to access virtual services. Additionally, mothers reported that continued weekly visits with their Home Visitor, even virtually, helped to mitigate the isolation resulting from social distancing and other public health restrictions. In doing so, the HIPPY delivery model embodies calls in the literature for holistic, highly contextualized social support that enables newcomers to address challenges effectively as they arise [5]. More broadly, this study contributes to the limited body of literature that examines the efficacy and impact of parent-centered home visiting interventions [68] with refugee populations during resettlement amid the COVID-19 pandemic [69].

Our study has several limitations. The study population was limited to refugee and refugee claimant mothers participating in the HIPPY program in Canada from September 2020 to June 2021. Of the 28 participating mothers, only two identified as current refugee claimants (others who arrived as claimants were permanent residents at the time of data collection), one as a single mother and two as living in an intergenerational household with older adults at the time of data collection. Therefore, this study does not represent the experiences of the refugee community in Canada. Most significantly, all of our participants were currently participating in the HIPPY program and engaging in weekly visits with their Home Visitor. As a result of frequent access to their Home Visitor and in light of our findings, the mothers in our study may have experienced improved access to resources and information from the onset of the pandemic, which may have mitigated the challenges experienced by other refugees and immigrant families. Finally, as part of our community-engaged research design, Home Visitors were present for interviews in their role as community researchers. While overall, we believe this to be a strength of our design, it is possible that the data were subject to social desirability bias.

Our findings can be used to inform future research and programming with refugee mothers and families both in the context of ongoing pandemic and post-pandemic recovery periods, as well as in efforts to increase preparedness for future social and economic shocks. There is little research on the experiences of refugee parents and families navigating resettlement in the context of the COVID-19 pandemic. While our research focused exclusively on the experiences of refugee mothers, there are few studies examining the experiences of refugee fathers, single mothers or refugee claimants exclusively in the Canadian context. Additionally, there is a dearth of research exploring the efficacy of various social intervention and models in terms of combating the challenges experienced by refugee families in Canada during the pandemic. Filling these research gaps would help to identify the needs of refugee groups and effective approaches to support families during resettlement. Our study suggests that technological and English literacy barriers influenced refugees' ability to pursue adult education, obtain access information and services and fulfill new parenting expectations in light of virtual, at-home learning. These barriers had implications for perceptions and experiences of mental wellbeing. Prioritizing technological literacy training during early resettlement is critical to mitigating ongoing and future barriers to education and health and social services as we shift toward increasingly standardized virtual models of service delivery. Innovations in the facilitation of virtual learning are critical to ensure inclusivity and accessibility for child and adult at-home learners in order to mitigate inequities in education, barriers to employment and an increased parental burden. These findings highlight the potential for parent-centered home visiting delivery models of social services as a tool to address the nuanced, family-specific challenges associated with refugee resettlement in Canada's diverse communities. Future research and pilot projects should be conducted to assess the efficacy of home visiting models in the contexts of a wide range of social services, as well as healthcare and health-centered service provision.

## 5. Conclusions

It is evident that challenges experienced by refugee mothers and families during resettlement have been amplified in the context of the COVID-19 pandemic. The pandemic has highlighted persistent weaknesses in the Canadian resettlement system that need to be addressed to ensure that mothers have access to the resources and support they require to meet their own mental health needs and feel confident and satisfied in their abilities to parent effectively. Moving forward, it is crucial to ensure that future research and program designs are founded on the objective of strengthening collective resilience [56]. In doing so, policymakers, service providers and other relevant stakeholders can reframe and recenter resettlement support systems and policies that are able to respond to refugee households' acute needs in the context of a prolonged crisis such as the pandemic. In turn, this will set a standard of care in which the onus to cope with adversity during resettlement does not solely rest with refugee mothers' and their families' ability to absorb shock as individuals and households [59],[60]. Instead, through holistic and responsive models of care, such as home-based parent-centered social interventions, a greater interconnectedness of families with wider support and systems can ensure that the brunt of social and economic shocks is absorbed by and overcome through, a unified and comprehensive system of resettlement support.
